# Clinicians' Assessment of Antisocial Personality Disorder (ASPD): A Network Analysis Approach on DSM‐5‐TR Criteria and Domains

**DOI:** 10.1002/pmh.70017

**Published:** 2025-05-07

**Authors:** Alessio Gori, Eleonora Topino, Carla Sharp

**Affiliations:** ^1^ Department of Health Sciences University of Florence Florence Italy; ^2^ Integrated Psychodynamic Psychotherapy Institute (IPPI) Florence Italy; ^3^ Department of Human Sciences LUMSA University of Rome Rome Italy; ^4^ Department of Psychology, 3695 Cullen Blvd., Room 126 University of Houston Houston Texas USA

**Keywords:** antagonism, antisocial personality disorder, detachment, disinhibition, lack of remorse, network analysis, personality disorder

## Abstract

Antisocial Personality Disorder (ASPD) is a personality disorder that entails significant impairments and/or costs at the individual, interpersonal, and community levels. Given its clinical relevance, scientific research is placing a significant focus on the study of the central characteristics of this condition to guide prevention and clinical practice. Within this framework, the present study aimed to investigate the associations and centrality of ASPD criteria and maladaptive trait domains in mental health professionals' conceptualization of the disorder, thus taking into account both categorical and dimensional approaches to personality pathology. The research involved 322 mental health professionals who reviewed the importance of ASPD criteria (Section II) and maladaptive trait domains (Criterion B of Section III). Data were analyzed using a network analysis approach. Both edge weights and node centrality were investigated. Within the criteria network, all centrality indices unanimously highlighted the role of lack of remorse. Regarding the trait domain network, the contributions of antagonism, detachment, and disinhibition were emphasized. The findings of this research collect and systematize the mental health professionals' perspective through the network analysis approach to provide further understanding of ASPD's central features. Such data may have useful practical implications for both research and clinical practice.

## 1 Introduction

### 1.1 Antisocial Personality Disorder (ASPD)

Antisocial Personality Disorder (ASPD) is classified as one of the 10 personality disorders in Section II of the fifth edition of the Diagnostic and Statistical Manual of Mental Disorders (DSM‐5; American Psychiatric Association 2013; American Psychiatric Association 2022), where it is described as “*a pervasive pattern of disregard for and violation of the rights of others*” (American Psychiatric Association 2022, *p*. 748). Additionally, it is one of the disorders listed in the Alternative Model of Personality Disorders (AMPD in section III; American Psychiatric Association 2013; American Psychiatric Association 2022) where, alongside problems in self‐ and interpersonal functioning, maladaptive personality traits such as manipulativeness, callousness, deceitfulness, hostility, risk‐taking, and impulsivity have been highlighted. While these traits, which belong to the domains of antagonism and disinhibition, are explicitly identified as key elements for diagnosis, the framework is further expanded by stating that “*trait and personality functioning specifiers may be used to record other personality features that may be present in antisocial personality disorder*” (American Psychiatric Association 2022, *p*. 886). This allows for exploring traits from other domains, such as Negative Affectivity, Detachment, and Psychoticism, thereby enriching the assessment process. Patients with ASPD tend to violate social norms and engage in criminal behaviors, resulting in overrepresentation within the judicial system and significant societal costs (National Institute for Health and Clinical Excellence 2010). ASPD is associated not only with the harm inflicted on others but also with elevated mortality rates, particularly in young individuals, largely due to reckless behaviors, accidents, or homicides (Yakeley and Williams 2014). Consistently, the scientific literature highlights high risks for individuals with ASPD to develop substance use disorders (Wojciechowski 2020; Gori et al. 2014) as well as behavioral addictions, such as gambling disorder (Lister et al. 2015). Furthermore, patients with ASPD commonly exhibit comorbidity with psychiatric disorders (see Hodgins et al. 2018 for a review), e.g., schizophrenia or affective disorders (Nichita and Buckley 2020), and significant rates of suicide (Krasnova et al. 2019). Given the clinical relevance of this personality disorder at the individual, interpersonal, and community levels, international scientific research has placed a significant focus on the study of the central characteristics of this condition to better inform treatment, but these efforts remain still not sufficient (see Black 2017; Yakeley and Williams 2014 for reviews). Within this framework, the network analytic approach could provide useful and relevant contributions.

### 1.2 Applying the Network Perspective on ASPD

The network analytic approach offers a powerful technique for investigating complex systems. Within this framework, nodes represent variables, individuals, or entities of interest, while edges denote their connections or interactions (Hevey 2018). Another key output in network analysis regards the measurement of centrality, which quantifies the relative influence of nodes within the network. Nodes with higher centrality are often considered more influential, as they are more connected and play a crucial role in facilitating interactions between other nodes (Roefs et al. 2022; Borsboom 2017). Network analysis has gained increasing interest as an approach to conceptualize psychopathology (Borsboom and Cramer 2013), by assuming that mental illness may be the result of the interaction between symptoms (i.e., the nodes) in a network (Cramer et al. 2010; Opsahl et al. 2010) or, more in general, for exploring inter‐relations con with other potentially associated aspects (Vierl et al. 2023; Vierl et al. 2024). This approach has also been used in the study of various personality disorders, such as avoidant (Marian et al. 2022) or borderline (Peters et al. 2023) personality disorders, suggesting the possibility that the condition is supported by the interactions among symptoms. Moreover, network analysis has been used in some research on psychopathy in both forensic patients (Preszler et al. 2018) and non‐institutionalized samples (Bronchain et al. 2019), in adults (Oba et al. 2024), young adults (Tsang and Salekin 2019), and adolescents (McCuish et al. 2019). However, although psychopathy shares some characteristics with ASPD, the disorders are conceptualized as distinguishable conditions (McKinley et al. 2018). Therefore, to the authors' knowledge, there are still no studies applying a network analysis approach to antisocial personality disorder. Furthermore, this approach has traditionally been used to assess interactions between observed symptoms (e.g., Vivarini et al. 2023). However, personality disorders have been described as ego‐syntonic conditions, in which maladaptive traits may not be experienced as internal conflicts and may be underestimated (Vinnars and Barber 2008). A person's access to their inner world is constantly influenced by several factors, including the degree of insight, self‐awareness, and the willingness to present themselves objectively (Huprich et al. 2011). This may be particularly relevant for ASPD, which is also characterized by a tendency to lie repeatedly and manipulate others (American Psychiatric Association 2013; American Psychiatric Association 2022). In line with this, previous evidence in the field has also shown that clinician assessments exhibited higher levels of concordance compared to self‐report measures (Gritti et al. 2016). Therefore, applying network analysis to the mental health professionals' evaluations of the most relevant elements for the diagnosis and the most representative features of ASPD, based on their overall clinical experience with this condition, may represent an innovative approach to identifying its most descriptive aspects.

### 1.3 The Present Research: The Mental Health Professionals' Perspective

At the time of writing, the network analysis approach has not yet been applied to ASPD for exploring the associations and the centrality of criteria and domains, according to the descriptions provided in the DSM‐5‐TR (American Psychiatric Association 2013; American Psychiatric Association 2022). Similarly, in existing studies on associated constructs and other personality disorders, various populations (i.e., clinical and community samples) have been involved, but research examining the view of mental health professionals is still lacking. To the best of the authors' knowledge, no studies have focused on the perspective of clinicians to investigate, using a network analysis approach, the representativeness of the symptoms for individuals diagnosed with ASPD. The application of the network analysis approach to mental health professionals' perceptions about the ASPD criteria and domains may have important implications for clinical practice. First, clinicians' evaluations are shaped by their cumulative experience, making them a valuable source of insight into how personality disorders manifest in real‐world settings. Furthermore, identifying the most representative elements of the disorder may provide important insights to support the diagnostic process, ensuring that the most representative and clinically useful aspects of the disorder are emphasized. Finally, identifying what clinicians consider to be the central and most representative aspects of ASPD and their associations may provide useful insights for treatment by indicating the most significant elements in strengthening or weakening the symptomatic network (Borsboom and Cramer 2013). To fill the aforementioned scientific literature gap and favor a nuanced understanding of how clinicians prioritize ASPD criteria and domains in real‐world practice, the present research aimed at exploring the relationships among the symptoms of ASPD, focusing on the perspective of a sample of mental health professionals. More precisely, based on the theoretical reference of the DSM‐5‐TR (American Psychiatric Association 2013; American Psychiatric Association 2022), the specific objectives of this study were:

1. to explore the associations and the centrality among the seven criteria of the ASPD, as described in section II (American Psychiatric Association 2013; American Psychiatric Association 2022);
2. to analyze the associations and the centrality among all the five maladaptive trait domains included in section III (American Psychiatric Association 2013; American Psychiatric Association 2022) for ASPD.

## 2 Method

### 2.1 Participants, Procedures, and Ethics

A sample of 322 mental health professionals (29.5% psychologists; 1.5% psychiatrists; 68.9% psychotherapists) was involved in this research (see Table 1). They were primarily women (77.3%), married (39.1%), or single (33.5%), and their ages ranged from 24 to 80 years (*M*_*age*_ = 42.31, *SD* = 12.25). Among the psychotherapists, different theoretical orientations were reported (see Table 1), primarily psychoanalytic (13.5%) or cognitive–behavioral (13.5%). Furthermore, most respondents reported having practiced clinical work for more than 10 years (46.9%). Participants were recruited through a snowball procedure starting from the researchers' contacts. To declare to be a licensed mental health professional and have a good command of the Italian language were the inclusion criteria. Furthermore, given the research topic, only clinicians who reported expertise in personality and personality disorders were allowed to complete the survey. This expertise was defined as having clinical experience with patients presenting such conditions, as well as theoretical knowledge of personality disorders as described by DSM‐5‐TR, with explicit reference to the chapters related to personality disorders in both Section II and Section III (American Psychiatric Association 2013; American Psychiatric Association 2022). The administration was via the Google Forms platform, and electronic informed consent was provided by each participant before starting the survey. All the procedures of this research were approved by the first author's institutional Ethical Committee.

**TABLE 1:** Demographic and professional characteristics of the mental health professionals involved in the research (*N* = 322).

Characteristics	M ± SD	*N* (*%*)
Age	42.31 ± 12.25	
Sex		
Males		73 (22.7%)
Females		249 (77.3%)
Marital status		
Single		108 (33.5%)
Married		126 (39.1%)
Cohabiting		64 (19.9%)
Separated		9 (2.8%)
Divorced		11 (3.4%)
Widowed		4 (1.2%)
Professional qualification		
Psychologist		95 (29.5%)
Psychiatrist		5 (1.6%)
Psychotherapist		222 (68.9%)
Theoretical model (for psychotherapists only)		
Psychoanalytic		43 (13.5%)
Psychodynamic		32 (9.9%)
Cognitive		26 (8.1%)
Cognitive Behavioral		43 (13.5%)
Humanistic		14 (4.3%)
Integrated		25 (7.8%)
Systemic		30 (9.3%)
Strategic		3 (0.9%)
Transnational		6 (1.8%)
Time exercising		
Less than a year		33 (10.2%)
1–2 years		19 (5.9%)
2–5 years		71 (22.5%)
5–10 years		48 (19.9%)
More than 10 years		151 (46.9%)

### 2.2 Measures

#### 2.2.1 Antisocial Personality Disorder Criteria—DSM‐5‐TR Section II (American Psychiatric Association 2013; American Psychiatric Association 2022)

Participants were requested to evaluate the significance of each criterion in influencing the diagnosis of ASPD, based on their overall clinical experience. Clinicians were specifically instructed to reflect on their cumulative professional knowledge and consider how each criterion typically impacts the diagnosis of ASPD in their practice. All criteria were listed as defined in DSM‐5‐TR Section II, and mental health professionals were asked to provide a rating for each one. Responses were scored on a 5‐point Likert scale, from 1 (*minimal significance*) to 5 (*great significance*).

#### 2.2.2 Antisocial Personality Disorder Domains—DSM‐5‐TR Section III (American Psychiatric Association 2013; American Psychiatric Association 2022)

Participants were requested to evaluate the representativeness of each *DSM‐5‐TR Section III* maladaptive trait domain for the disorder, based on their overall clinical experience. Clinicians were specifically instructed to reflect on their cumulative professional knowledge and evaluate how well each domain reflects the core characteristics of ASPD in their clinical observations. Assuming that the mental health professionals have a good knowledge of maladaptive trait domains, facets were not included to simplify the survey completion process. All domains were listed as defined in DSM‐5‐TR Section III, and mental health professionals were asked to provide a rating for each one. Responses were scored on a 5‐point Likert scale, from 1 (*not at all representative*) to 5 (*very representative*).

### 2.3 Analytic Plan

The JASP (Jeffrey's Amazing Statistics Program, v. 0.19.1; JASP Team 2023) software for Windows was used to analyze data. Descriptive statistics have been calculated to provide an overview of the study variables and information about their distribution. An absolute skewness value of 2 or less and an absolute kurtosis value of 7 or less were considered indicative of a normal distribution (Kim 2013). The criteria and domain networks for Antisocial Personality Disorder were analyzed separately. For each investigation, a regularized partial correlation network (also known as a Gaussian Graphical Model) was estimated following the EBICglasso procedure. This method employs the graphical Least Absolute Shrinkage and Selection Operator (GLASSO) (Friedman et al. 2008) regularization based on the Extended Bayesian Information Criterion (Chen and Chen 2008), ensuring that the model optimally balances complexity and goodness of fit. The hyperparameter (γ) for the GLASSO, which determines the degree of penalization applied to the associations, was set to 0.5, as suggested by previous literature (Friedman et al. 2008). This value controls the sparsity of the network, ensuring that the associations retained are interpretable while minimizing the risk of overfitting. For the network estimation, the “Auto” option was set concerning the correlation method, which automatically detects the variable type and uses the most suitable correlation type. The visualization of the networks was generated using the force‐driven Fruchterman–Reingold algorithm (Fruchterman and Reingold 1991) to provide an interpretable and stable layout for both networks. Each network consists of nodes (the observed variables, i.e., the criteria or the domains) and edges (the relationships between the nodes within the network system) (Burger et al. 2023). First, the interpretation of the global network structure was guided by effect size thresholds for practical relevance, with edge weights of at least ≥ 0.2 indicating potential practical significance (Ferguson 2016). To test the structural importance of each criterion or domain in the networks, three indices of node centrality were estimated (Borsboom 2017; Morey et al. 2022): (1) *Betweenness* quantifies how often a node lies on the shortest path connecting any two other nodes; (2) *Closeness* indicates how close a node is to all other nodes within the network, suggesting its effectiveness in spreading information throughout the network; (3) *Strength* is the sum of the absolute edge weights that a node shares with all other nodes in the network. Case‐dropping bootstrapping (1000 times) with 95% confidence intervals was used to evaluate the stability of the networks. This method examines whether centrality indices remain consistent after re‐estimating the network with a reduced number of cases. A stability coefficient of at least 0.25 is considered desirable, preferably surpassing 0.5 (Morey et al. 2022; Epskamp et al. 2018).

## 3 Results

Demographic and professional characteristics of mental health professionals are presented in Table 1. Table 2 provides descriptive statistics for the variables. The sample distribution is considered approximately normal, as evidenced by absolute skewness and kurtosis values below 2 and 7, respectively.

**TABLE 2:** Descriptive statistics of the study variables.

	Mean	Std. deviation	Skewness	Kurtosis	Minimum	Maximum
Criterion 1	4.205	0.897	−1.118	1.072	1	5
Criterion 2	4.062	0.901	−0.996	1.021	1	5
Criterion 3	3.599	1.076	−0.539	−0.234	1	5
Criterion 4	3.898	0.947	−0.770	0.370	1	5
Criterion 5	4.059	0.944	−0.879	0.336	1	5
Criterion 6	3.624	0.960	−0.443	−0.045	1	5
Criterion 7	4.419	0.832	−1.633	2.912	1	5
Domain 1	3.696	0.992	−0.475	−0.244	1	5
Domain 2	3.960	0.968	−0.604	−0.529	1	5
Domain 3	3.792	0.994	−0.492	−0.374	1	5
Domain 4	3.643	1.017	−0.491	−0.326	1	5
Domain 5	3.146	1.130	−0.056	−0.704	1	5

*Note:* Criterion 1 = Failure to conform; Criterion 2 = Deceitfulness; Criterion 3 = Impulsivity; Criterion 4 = Aggressiveness; Criterion 5 = Reckless disregard; Criterion 6 = Irresponsibility; Criterion 7 = Lack of remorse. Domain 1 = Negative Affectivity; Domain 2 = Detachment; Domain 3 = Antagonism; Domain 4 = Disinhibition; Domain 5 = Psychoticism.

### 3.1 Global Network Structure

**FIGURE 1:**
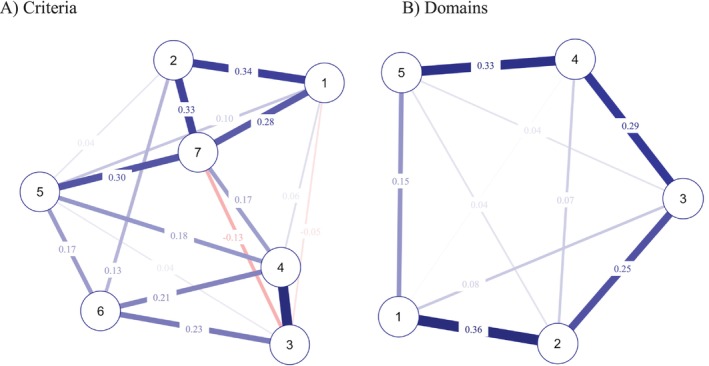
The network plots of criteria (A) and domains (B) for the antisocial personality disorder. *Note:* Red lines show negative associations between criteria, while blue lines represent positive relationships between them. Thicker lines indicate stronger edge weights. Criterion 1 = Failure to conform; Criterion 2 = Deceitfulness; Criterion 3 = Impulsivity; Criterion 4 = Aggressiveness; Criterion 5 = Reckless disregard; Criterion 6 = Irresponsibility; Criterion 7 = Lack of remorse. Domain 1 = Negative Affectivity; Domain 2 = Detachment; Domain 3 = Antagonism; Domain 4 = Disinhibition; Domain 5 = Psychoticism.

The network of criteria for antisocial personality disorder consisted of seven nodes, and 17/21 edges were non‐zero (see Figure 1A). Within the network, the strongest connection was observed between criterion 3 (Impulsivity) and criterion 4 (Aggressiveness). Additionally, a significant association was found between criterion 1 (Failure to conform) and criterion 2 (Deceitfulness), as well as between criterion 4 (Aggressiveness) and criterion 6 (Irresponsibility). The node showing the most significant associations was that related to lack of remorse (criterion 7), specifically with criterion 1 (Failure to conform), criterion 2 (Deceitfulness), and criterion 5 (Reckless disregard).

The network of maladaptive trait domains for antisocial personality disorder consisted of five nodes, and 10/10 edges were non‐zero (see Figure 1B). Within the network, the strongest connection was observed between domain 1 (Negative Affectivity) and domain 2 (Detachment). Significant associations were found between domain 2 (Detachment) and domain 3 (Antagonism), between domain 3 (Antagonism) and domain 4 (Disinhibition), as well as between domain 4 (Disinhibition) and domain 5 (Psychoticism).

**FIGURE 2:**
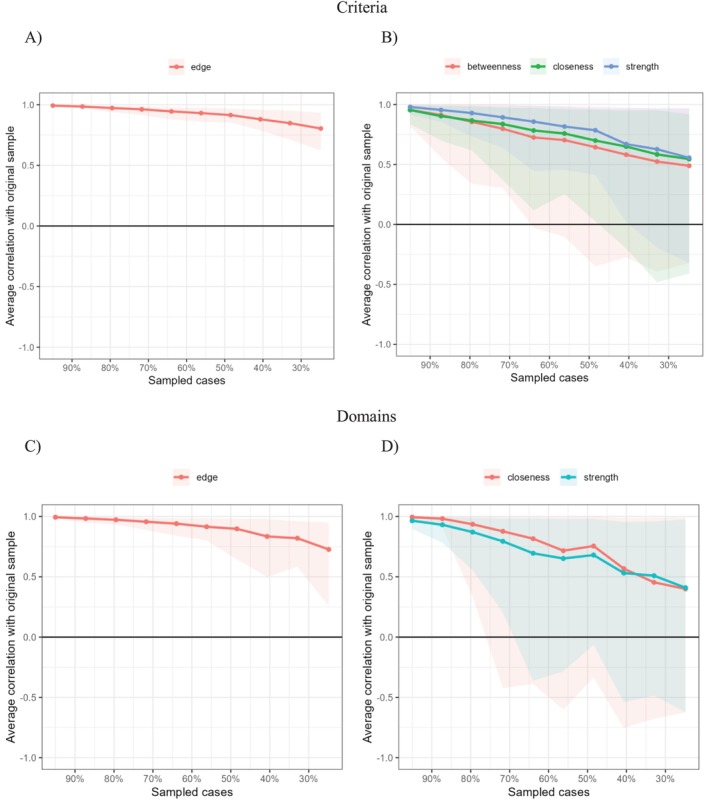
Stability of the edge‐weight estimates (A and C) and centrality indices (B and D) based on bootstrap. *Note*: Areas indicate 95% CI.

The bootstrap analysis supported the stability of the edge‐weight estimates (see Figure 2A,C).

### 3.2 Indices of Centrality

The centrality measures are shown in Table 3.

**TABLE 3:** Centrality measures for criteria and domains for the antisocial personality disorder.

Criteria	*B*	*C*	*S*	Domain	*B*	*C*	*S*
Criterion 1	−0.601	−1.073	−0.503	Domain 1	0.000	−0.923	−0.803
Criterion 2	−0.134	−0.122	−0.414	Domain 2	0.000	0.017	**1.045**
Criterion 3	−0.601	−0.926	−0.174	Domain 3	0.000	**1.593**	0.189
Criterion 4	−0.134	0.440	0.849	Domain 4	0.000	0.088	0.817
Criterion 5	−0.134	0.741	−0.552	Domain 5	0.000	−0.776	−1.249
Criterion 6	−0.601	−0.711	−1.053	—	—	—	—
Criterion 7	**2.205**	**1.651**	**1.847**	—	—	—	—

*Note:* For each index, the bold indicate the highest value. B = Betweenness; C = Closeness; S = Strength. Criterion 1 = Failure to conform; Criterion 2 = Deceitfulness; Criterion 3 = Impulsivity; Criterion 4 = Aggressiveness; Criterion 5 = Reckless disregard; Criterion 6 = Irresponsibility; Criterion 7 = Lack of remorse. Domain 1 = Negative Affectivity; Domain 2 = Detachment; Domain 3 = Antagonism; Domain 4 = Disinhibition; Domain 5 = Psychoticism.

Concerning the criteria, Criterion 7 (lack of remorse) appears central in the network for all three indices (betweenness, closeness, and strength) for the antisocial personality disorder. The bootstrap analysis supported that the node centrality was stable and interpretable in this network (see Figure 2B).

Since the domains are all interconnected (10/10 non‐zero edges) the direct paths between the nodes are the shortest ones. None of the shortest paths pass through intermediate nodes, and therefore the betweenness index was zero for all the nodes. Concerning closeness, the node with the highest values was domain 3 (Antagonism). Furthermore, the node showing the highest strength was domain 2 (Detachment), followed by domains 4 (Disinhibition) and 3 (Antagonism). The bootstrap analysis confirmed that the two indices are stable (see Figure 2D).

## 4 Discussion

Antisocial Personality Disorder (ASPD) is characterized by externalizing manifestations, sometimes linked to criminal behaviors, which may have a significant impact on individuals afflicted by it, those who interact with them, and society (see Mark et al. 2022 for a review). Given its clinical relevance, a substantial body of research has been dedicated to furthering understanding of this condition to inform therapeutic strategies. Consistent with this objective, the present study aimed to explore the associations and centrality among the symptoms of ASPD, drawing on mental health professionals' viewpoints. Therefore, the Network Analysis Approach was employed to provide both visual and quantitative insights into the criteria or domains characterizing the disease, as well as their interconnections within the two networks. This approach may offer valuable suggestions for understanding the rationale implicitly or explicitly employed by mental health professionals in diagnosis, with consequent repercussions on treatment conceptualization (see Cramer et al. 2010 for a review).

### 4.1 Relationships Among the Criteria

The ASPD criteria form a network with numerous interconnections among symptoms. Specifically, the most robust connection was observed between impulsivity (criterion 3) and aggressiveness (criterion 4). In other words, mental health professionals perceive these aspects as closely associated within their diagnostic evaluations of the disorder. Specifically, when clinicians rated aggressiveness as more significant for ASPD, they also tended to consider impulsivity as a representative criterion. This perception may reflect the observation that impulsivity in ASPD frequently correlates with externalizing and aggressive behaviors, as highlighted in recent evidence (Mark et al. 2022). Furthermore, the lack of remorse (criterion 7) showed the highest number of connections, particularly exhibiting the strongest associations with failure to conform (criterion 1), deceitfulness (criterion 2), and reckless disregard (criterion 5). This pattern suggests that clinicians do not merely observe these criteria as isolated elements but rather perceive “lack of remorse” as a central feature that draws their attention during the evaluation of ASPD and connects to the assessment of other maladaptive behaviors associated with this diagnosis. This connection reflects the clinical reality where such behaviors often co‐occur, and it supports the notion that “lack of remorse” serves as a foundational criterion in the disorder's manifestation. Indeed, previous studies indicated that the presence of guilt and shame typically correlates with a higher inclination towards ethical conduct (Martin et al. 2019). Conversely, the lack of remorse in individuals with ASPD has been shown to predict sustained aggressive and dangerous behaviors towards themselves and others, including bullying, threats or intimidation, physical altercations, use of dangerous weapons, and acts of cruelty (Cohen 2010; Goldstein et al. 2006). Consistently, the lack of remorse criterion also exhibited the highest node–network association, with values aligned across all three indices (betweenness, closeness, and strength). Such data suggests that mental health professionals perceive lack of remorse as the basic criterion for diagnosing ASPD, which may influence all other features of the disorder. In line with this, indeed, several authors have highlighted the importance of assisting patients with ASPD in recognizing their responsibilities, avoiding colluding with minimization, and with the tendency to externalize by blaming others (Piquero 2017), considering this as one of the core elements of therapy with patients having this personality disorder (Gabbard 2021; Aerts et al. 2023).

### 4.2 Relationships Among Maladaptive Trait Domains

The network pertaining to the AMPD domains as applied to ASPD appears dense, as it features nodes all interconnected with each other, consistently with previous evidence showing high inter‐domain correlations (Gibbon et al. 2020). This pattern suggests that clinicians perceive the maladaptive traits associated in ASPD as highly interconnected. Although some dimensions may tend to predominate over others, potentially giving rise to different subtypes (see Anderson and Kelley 2022; McKinley et al. 2018 for reviews), the lack of sparsity in the network reflects the mental health professionals' view that these traits co‐occur frequently, rather than existing as isolated characteristics. These insights are further enriched by exploring centrality indices. In this regard, antagonism emerges as the node closest to all other domains within the network, thus indicating its efficacy in influencing the others (*Closeness* centrality index). These findings are also consistent with a previous comprehensive meta‐analytic investigation using the Five Factor Model (FFM) domains, in which antagonism (i.e., low agreeableness) was shown to be the primary correlate of antisocial behavior outcomes (Vize et al. 2019). Furthermore, detachment and disinhibition emerge as the nodes having the higher number of connections within the network, thus indicating its efficacy in influencing the whole condition (*Strength* centrality index). This result was partially expected and predictable. Previous evidence has indeed identified high levels of impulsivity and low response inhibition as distinctive features of ASPD (e.g., Swann et al. 2009). On the other hand, the data have also highlighted the role of detachment, which unlike the other two domains identified by centrality indices (i.e., antagonism and disinhibition), is not listed among the features indicated for ASPD in the DSM‐5 (American Psychiatric Association 2013; American Psychiatric Association 2022). Given that the majority of studies that have identified antagonism and disinhibition as central features of Section III ASPD are based on self‐report (Anderson and Kelley 2022), the addition of detachment as identified by mental health professionals raises interesting questions. It is plausible that mental health professionals have emphasized the centrality of detachment with specific reference to the traits of emotional coldness and intimacy avoidance observed in some subtypes of individuals with ASPD (Marsden et al. 2019; Yoon and Knight 2015), aligning with findings that demonstrate an association between detachment, aggressiveness, and hostility (Lynam and Miller 2019). However, this interpretation is speculative and requires further investigation, particularly to explore how subtypes and comorbidities may influence clinicians' evaluations of ASPD domains.

### 4.3 Practical Implications

The international scientific community has highlighted the need for further theoretical and practical efforts concerning the study of the treatment for ASPD (see Black 2017 for a review). In light of these shortcomings, the use of innovative approaches such as network analysis can be particularly functional in contributing to advancing research and, consequently, clinical practices in this field. Specifically, the results of the present study provide valuable insights into how mental health professionals conceptualize ASPD criteria and domains, offering practical implications for therapy and diagnosis. From a network modelling perspective, which focuses on analyzing multivariate relationships between symptoms and domains (see Isvoranu et al. 2022 for a more in‐depth understanding), the results suggest the significance of considering lack of remorse as a central node, closely connected to failure to conform, deceitfulness, and reckless disregard for safety. Moreover, the domains of antagonism, detachment, and disinhibition emerge as key dimensions shaping clinicians' representations of ASPD. These findings suggest that clinicians perceive these dimensions as particularly influential and likely to co‐occur with other criteria and domains, reflecting their cumulative experience with patients diagnosed with ASPD. Identifying these perceived connection patterns could support the diagnostic process and guide targeted therapeutic interventions. Further insights could be drawn by embracing the network theory of psychopathology (Cramer et al. 2010), according to which each syndrome can be conceptualized as driven by the interaction among symptoms, which are considered “*constitutive of mental disorders, not reflective of them*” (McNally 2016, *p*. 95). Therefore, identifying the most salient domains and criteria can be beneficial for understanding which aspects might influence the whole network (see Cramer et al. 2010 for a review). Although this perspective has been effectively applied to various mental health disorders (Borsboom et al. 2011; Cramer et al. 2016), the underlying premise is that bidirectional causal interactions exist within the network. Therefore, while the theoretical base supports these types of associations, these data should be interpreted with caution, and future time‐series data should be employed to confirm such inferences. Within this conceptual framework, lack of remorse (criterion), along with antagonism, detachment, and disinhibition (domains), could be pivotal markers for clinicians in the diagnostic process. These central aspects may assist clinicians in evaluating other connected criteria, contributing to a more comprehensive understanding of the disorder.

### 4.4 Limitations and Suggestions for Future Research

A few limitations of this study should be noted. Firstly, while this study made use of Section III hybrid diagnosis of ASPD in addition to Section II criteria, it did not utilize a full dimensional approach to personality pathology. Doing so would have required consideration of Criterion A of the AMPD (Level of Personality Functioning) and all Criterion B traits. In this sense, by focusing on the hybrid diagnosis of ASPD, the current study still promotes the idea that personality disorders are delimited from one another. However, there are significant cross‐loadings of personality disorder criteria across underlying personality disorder categories, suggesting that personality disorders may be better conceptualized as a general factor representing the shared features of all personality disorders (Sharp et al. 2015; Wright and Simms 2016). Secondly, although the network analysis theory of psychopathology (Cramer et al. 2010) assumes a causal bidirectional relationship among the symptoms that constitute the condition, it is important to exercise caution in interpreting these associations. The clinician‐rated covariances observed in this study reflect mental health professionals' aggregated evaluations and perceptions based on their cumulative experience, and theory‐driven interpretations can provide insights into how clinicians perceive the interaction among the most representative ASPD symptoms; however, it must be acknowledged that the cross‐sectional design of this study inherently limits causal inference. These findings may be enriched in future research employing time‐series data or longitudinal designs, with data about specific patients collected over time and repeated assessments. Such approaches would allow for observing variations in mental health professionals' evaluations, for example, during targeted interventions or in association with changes in other factors (e.g., therapeutic alliance, clinical expertise, professional self‐efficacy; Gori et al. 2023; Gori et al. 2022). This would provide stronger evidence for causal interpretations and enable a more detailed exploration of the dynamic nature of symptom networks. Third, antisocial personality disorder has been investigated in line with the concepts of the DSM‐5‐TR (American Psychiatric Association 2013; American Psychiatric Association 2022), without the exploration of specific subtypes and comorbidities that may be encountered in clinical practice. Future research endeavors should strive in this direction in a more in‐depth and accurate way, to further enhance the understanding of the heterogeneity within ASPD and inform more tailored treatment approaches. Lastly, the data solely relies on clinician reports, which, while dependable, may not entirely capture the perspectives and experiences of individuals with ASPD. Specifically, the network theory of psychopathology (Cramer et al. 2010) has traditionally been applied to investigate causal relationships between symptoms as observed by patients. The application of this framework to clinicians' perspectives represents a methodological shift, as it relies on their aggregated professional experience with ASPD rather than observations from individual patients. This approach assumes that mental health professionals, when evaluating the significance of criteria and domains, inherently draw upon their cumulative knowledge and exposure to a wide range of cases, reflecting the most representative symptoms of AMPD. Placing these elements within a network framework allows for a deeper understanding of the associations between them, as conceptualized by clinicians. Although this new methodology offers certain advantages (e.g., considering the clinician's overall experience, bypassing potential issues such as the patient's lack of insight or tendency to deceive), it still requires further validation and exploration. For future research, incorporating multiple sources of information, such as self‐reports from individuals with ASPD, collateral reports from family members or peers, and objective measures, could provide a more comprehensive and nuanced understanding of the disorder from various perspectives. Additionally, qualitative studies exploring the subjective experiences of individuals with ASPD could offer valuable insights into their lived experiences and treatment needs.

## 5 Conclusions

This study is the first to use a network analytic approach to examine associations and centrality of ASPD criteria and domains, based on the mental health professionals' understanding of the disorder. The findings highlighted the centrality of lack of remorse among the criteria, and antagonism, detachment, and disinhibition among the domains. These results may have useful practical implications by enriching the existing research on ASPD and supporting clinical practice, providing insight into both the diagnostic and treatment phases.

## Author Contributions

**Alessio Gori:** conceptualization (lead); methodology (equal); formal analysis (equal); data curation (lead); writing – original draft preparation (equal); writing – review and editing (equal); supervision (lead). **Eleonora Topino:** methodology (equal); formal analysis (equal); data curation (equal); writing – original draft preparation (equal); writing – review and editing (equal). **Carla Sharp:** writing – review and editing (equal); supervision (supporting). All authors have read and agreed to the published version of the manuscript.

## Ethics Statement

The research was approved by the Ethical Committee of the Integrated Psychodynamic Psychotherapy Institute.

## Consent

Informed consent was obtained by all the subjects involved in the research.

## Conflicts of Interest

The authors declare no conflicts of interest.

## Data Availability

The data presented in the present study are available upon reasonable request from the corresponding author.
